# Dynamic Mitigation Mechanisms of Rime Icing with Propagating Surface Acoustic Waves

**DOI:** 10.1021/acs.langmuir.2c01509

**Published:** 2022-09-07

**Authors:** Deyu Yang, Luke Haworth, Prashant Agrawal, Ran Tao, Glen McHale, Hamdi Torun, James Martin, Jingting Luo, Xianghui Hou, YongQing Fu

**Affiliations:** † Faculty of Engineering, University of Nottingham, Nottingham NG7 2RD, U.K.; ‡ Faculty of Engineering and Environment, Northumbria University, Newcastle upon Tyne NE1 8ST, U.K.; § Shenzhen Key Laboratory of Advanced Thin Films and Applications, College of Physics and Optoelectronic Engineering, Shenzhen University, Shenzhen 518060, China; ∥ School of Engineering, University of Edinburgh, Edinburgh EH9 3JL, U.K.

## Abstract

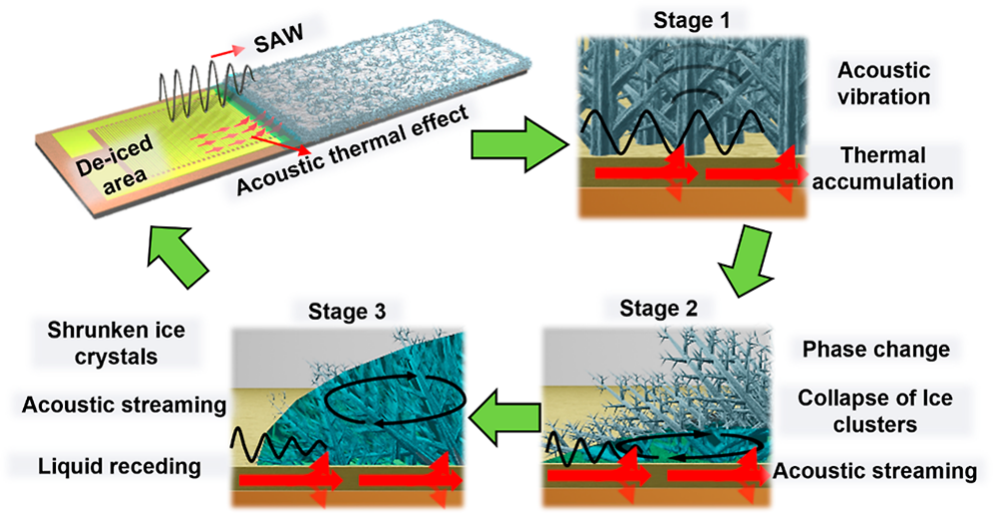

Ice accretion on economically valuable and strategically important surfaces poses significant challenges. Current anti-/de-icing techniques often have critical issues regarding their efficiency, convenience, long-term stability, or sustainability. As an emerging ice mitigation strategy, the thin-film surface acoustic wave (SAW) has great potentials due to its high energy efficiency and effective integration on structural surfaces. However, anti-/de-icing processes activated by SAWs involve complex interfacial evolution and phase changes, and it is crucial to understand the nature of dynamic solid–liquid–vapor phase changes and ice nucleation, growth, and melting events under SAW agitation. In this study, we systematically investigated the accretion and removal of porous rime ice from structural surfaces activated by SAWs. We found that icing and de-icing processes are strongly linked with the dynamical interfacial phase and structure changes of rime ice under SAW activation and the acousto-thermally induced localized heating that facilitate the melting of ice crystals. Subsequently, interactions of SAWs with the formed thin water layer at the ice/structure interface result in significant streaming effects that lead to further damage and melting of ice, liquid pumping, jetting, or nebulization.

## 1 Introduction

Ice accretion on structural surfaces is one of the critical hazards in aerospace, power transmission, offshore platform, and wind turbine sectors.^1−4^ Based on the ice’s morphology and density, there are two major hazardous ice types: rime ice and glaze (or clear) ice. Compared to glaze ice, the formation of rime ice is mainly regarded as an instant surface freezing process of supercooled water droplets, with features such as low liquid water contents (generally lower than 0.1 g/m^3^), strong supercooling/low temperatures (generally lower than −10 °C), and porous (e.g., loose with a high air-filled porosity) and cluster shapes after freezing.^5,6^ The formation of porous structures is mainly due to the quick freezing of the supercooled droplets without residual liquid water that fills the gaps. Under low-humidity conditions and at subzero temperatures, supercooled droplets are easily deposited on the structural surfaces and then gradually form a thick layer of rime ice.^6,7^

Commonly applied ice mitigation technologies include both passive approaches (such as the use of icephobic surfaces^8^) and active techniques (such as electro-impulsive/expulsive, resistance heating, hot-air bleeding, ultrasonic methods, and chemical fluids^9−13^). However, their efficiency and sustainability for ice protection have significant limitations. For example, icephobic surfaces often have issues of poor mechanical or long-term durability.^8,14^ The chemical fluids used for the removal of accreted ice could cause severe environmental issues.^15,16^ The electrical heating method often consumes excessive energy for ice prevention or removal. Therefore, innovative ice mitigating techniques with high energy efficiency and environment-friendly features are critically required.

Surface acoustic wave (SAW) technologies have been widely applied in wireless communications, acoustofluidics, sensors, particle/cell concentrating, and micro-heaters.^17−21^ Multiple wave modes (including Rayleigh, Lamb, Love, and shear horizontal SAWs) and their hybrid waves can be generated and then propagate along the structural surfaces.^22,23^ Compared to the conventional bulk piezoelectric material-based SAW devices, thin-film-based SAWs have the advantage that they can integrate multiple functions into a single structure on different substrates, such as silicon, metals, glass, or polymers.^24^ Besides the wide applicability on various substrate materials, SAWs can be generated and then propagate on surfaces with different features, even on flexible and bendable surfaces if thin film technology is used.^25^ Furthermore, the direction of propagating SAWs can be designed across the whole solid surfaces.^26^ Acoustofluidic phenomena generated using thin-film SAW devices, including liquid mixing, transportation, jetting, nebulization, droplet generation, and particle/biological cell sorting and manipulations, have recently been reported.^27−30^

In the field of ice mitigation, thin-film-based SAWs have already been demonstrated to effectively generate both acoustic wave vibration and thermal effects on the device surface, thus offering great potentials for both anti-icing and de-icing with a high efficiency.^31,32^ However, interfacial behaviors, ice removal, and prevention mechanisms for both anti-icing and de-icing of the rime ice under propagating SAWs have never been explored. Compared with conventional acoustofluidic research of sensors or laboratory-on-a-chip using the thin-film SAW devices,^24,27,33,34^ ice protection and mitigation using thin-film SAWs are more complex, mainly because there is varied phase evolution (from solid, liquid, to vapor, or their mixtures) and dynamic evolution of interfacial microstructures during the processes under the agitation of propagating waves. There is a lack of in-depth investigations on the interfacial responses and phase evolution driven by SAWs during the icing and de-icing processes, which restricts the further exploration of SAW devices for ice mitigation.

This study is focused on the anti-icing and de-icing mechanisms of porous rime ice on a structural surface (aluminum plates), integrated with ZnO-based thin-film SAWs. We first investigate the fundamental issues about interactions of SAWs with the rime ice and/or liquid/ice mixtures. Then, we focus on the experimental studies of anti/de-icing performance for rime ice using thin-film SAW devices. Finally, the evolution of ice morphology and phase changes at different humidity levels under icing conditions and different SAW powers are investigated, from which the de-icing and anti-icing mechanisms using thin-film SAWs are verified.

### 1.1 Anti-Icing and de-Icing of Rime Ice with SAWs

**Figure 1:**
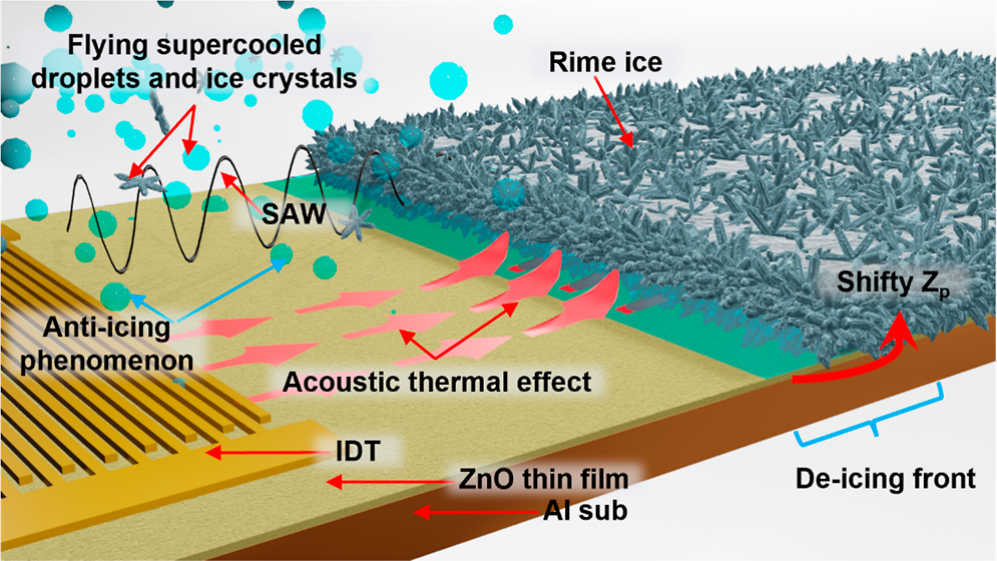
Schematic illustration of interfacial behaviors between the rime ice and SAW device surface.

Nanoscale surface wave vibrations (induced by the propagating SAWs from the surface into ice or liquid) and the acoustic thermal heating effect are two main mechanisms for ice protection using SAW devices.^31^ They play key roles in the anti/de-icing process by preventing ice accumulation and effectively removing the formed ice.^31^ In this section, we address this issue by considering the phase evolution of ice, water, and vapor and connect them with anti/de-icing mechanisms with SAWs. The hypotheses about the anti/de-icing processes and mechanisms are first established and then proved by the designed experiments. The whole process can be considered in three different configurations with the presence of (1) solid and dry porous rime ice, (2) the ice/liquid mixture (i.e., some of the ice is partially melted), and (3) the liquid water/water vapor stage (changed into liquid and gradually evaporated). Figure 1 illustrates the conceptional anti/de-icing processes in the presence of rime ice on the SAW device in a humid and frozen environment.

### 1.2 SAW-Induced de-icing Process

#### 1.2.1 Stage One: Solid and Porous Rime Ice

At the first stage, where only solid and porous rime ice crystals exist, the key phenomena at the interface are the propagation of SAWs on the device’s surface, mainly through the interface between the device and the porous rime ice. We believe that the acoustic impedance of the porous rime ice is a key parameter that affects the propagation of SAWs along the surface of the structure. Currently, there are different theoretical models (such as Biot’s theory and Pyett’s theory) proposed to investigate the acoustic impedance of snow or rime ice with various densities.^35−39^ Most of these models were based on two assumptions, that is, the acoustic waves have relatively low frequencies (from hundred to thousand Hz); and the rime ice is a rigid-frame model with a stable structure. However, the structure of real rime ice is highly porous and fragile. To ascertain the transmission of SAW energy from the substrate to the porous rime ice, we use the Johnson–Champoux–Allard (JCA) fluid (such as air) model to determine the acoustic impedance of the porous layer (*Z*_p_)^40−44^
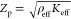
1where
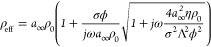
2
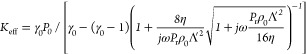
3where *a*_*∞*_ and ϕ are the tortuosity and porosity of the porous layer, respectively, η and ρ_0_ are the viscosity and density of the fluid (e.g., air) in the porous layer, respectively, ω is the angular frequency of the wave, *Pr* is the Prandtl number, σ is the flow resistivity of the porous layer, Λ and Λ′ are the viscous characteristic length and the thermal characteristic length of the porous layer, respectively γ_0_ is the ratio of specific heats, and *P*_0_ is the static pressure.

Based on the above background information, Biroun et al.^45^ reported that there is a large impedance mismatch between the surface of the SAW devices along with the wave propagation and the porous superhydrophobic nanoparticle layer coating (ϕ ≈ 0.57), which results in a wave reflection coefficient of 0.998, calculated using
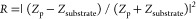
4

This low wave reflection coefficient indicates a weak transmission of acoustic energy at the substrate–porous layer interface. Although it is challenging to quantify the porosity of the rime ice layer, we can reasonably assume that the porosity of the rime ice will be higher than that of the nanoparticle porous coating in ref (45). Therefore, in our case, the SAW acoustic energy will not easily be transmitted through the rime ice layer; thus, the energy will be dissipated significantly into the interfacial layer and the rime ice layer. Nanoscale vibrations induced by SAWs easily break the roots of the rime ice clusters, thus changing the structure of porous rime ice and surface ice morphology.

Apart from the significant surface vibrations, the applied radio frequency power also induces a localized heating effect (also called the acoustic thermal effect) in the thin-film SAW devices due to the SAW energy dissipation. This thermal effect is generated by the high-frequency-induced mechanical vibration and stress generated in the substrate.^24^ The heating effect then conducts the viscous dissipation of the acoustic energy into the liquid such as a sessile droplet, as extensively reported in refs^20^ and^46^ –^47^^48^^49^.

#### 1.2.2 Stage 2: The Solid Ice Crystals Coexist with Liquid

With the activation of SAW energy, the interfacial ice layer is partially melted, thus forming the melted liquid at the interface due to the phase evolution. Once the liquid phase starts to appear at the device/ice interface, the de-icing front at the interface (see Figure 1) will be changed from a porous but rigid rime ice into an ice–liquid water mixture, which results in a decrease in acoustic impedance. At the interface between water and the substrate, if we assumed a reflection coefficient of 0.7 (which is linked with the situation when ice becomes melted), the SAW energy transmitted from the substrate is estimated to increase from 0.2% (with porous rime ice) to 30% (liquid water). Combined with the substrate thermal heating effect, absorption of SAW energy by the melted liquid significantly enhances the exchange of mass and heat inside the liquid due to the internal streaming and liquid flow. The wave–liquid interaction is driven by the SAW streaming force, *F*_*s*_, which is given using the following expression^50−52^
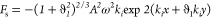
5where ϑ_1_ = *j*ϑ (with *j*^2^ = −1) is the attenuation constant and  and ν_L_ and υ_S_ are the wave velocities in the liquid and on the solid surface, respectively. *A* is the wave amplitude, ω is the angular velocity, *k* is the wave number of the leaky SAW, and subscripts *i* and *j* = 1, 2, and 3 represent the *x*, *y*, and *z* coordinates. Sudeepthi et al.^53^ reported the transition of wetting behaviors on a porous and nanoparticulate surface with the application of SAWs. They observed that with the agitation of SAWs, the state of surface droplets was changed from a Cassie state to a Wenzel state, indicating that SAW agitations caused the surface water to penetrate into the porous layer. Therefore, this indicates that the SAW streaming force can effectively prompt water penetration and propagation into the porous rime ice layer and also significantly enhance the acoustic thermal effect.

Under the activation of propagating SAWs, this de-icing front quickly moves along the SAW propagating direction, away from the IDT area. With the continued de-icing process, the porous rime ice will shrink gradually until it is changed into multiple independent ice crystals flowing with the internal streaming induced by SAWs. In this case, the semi-melted ice crystals dispersed in the liquid are governed by the acoustic radiation force (*F*_*R*_) and acoustic streaming drag force (*F*_*d*_), which are^54−56^
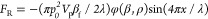
6
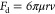
7where *p*_0_ is the acoustic pressure, *V*_p_ is the volume of the ice crystals, β and ρ are the compressibility and density of the melted liquid and ice crystals, respectively, φ is the acoustic contrast factor, λ is the wavelength of the acoustic waves, *x* is the distance from the pressure node, and μ, *r*, and *v* are the dynamic viscosity of the liquid, radius, and relative velocity of the ice crystals, respectively. The significant flowing of ice crystals can accelerate their elimination and enhance the mass changes inside the liquid.

In brief, after the occurrence of phase changes from solid ice to liquid, the exchange of mass and heat within the rime ice is enhanced by the acoustic pressure and agitation, which effectively promotes the de-icing process.

#### 1.2.3 Stage 3: Melted Liquid Activated by SAWs

When the ice crystals are completely melted and transformed into a liquid state, the acoustic wave will induce different effects, depending on the applied power. The waves may either drive the liquid away, nebulize the liquid into mists at high power levels, or the generated heat can quickly evaporate the liquid layer from the surface.^24^

### 1.3 Anti-Icing Mechanisms for Rime Ice

Based on the theory of homogeneous ice nucleation in the air, supercooled droplets would be the main phase that flies and is attached to the solid surface and then nucleates, grows, and forms the porous rime ice due to the low temperature nucleation centres.^6,57^ However, considering the possible particles in the air (such as dust), some ice crystals may also be initiated in the gas phase from the water vapor. Thus, in this study, both supercooled droplets (the main phase) and ice crystals (the secondary phase) will be discussed in the anti-icing process.

For the newly attached supercooled droplets that are generated in the subzero environment, the SAWs prevent ice nucleation and accretion by restricting the size of ice embryos to be smaller than the critical nucleolus radius, *r*_c_, and increasing the critical free energy of heterogeneous ice nucleation, ΔG*, which has been reported in ref (31). The attached supercooled droplets are also affected by vibration and thermal effects. This hybrid effect offers an advanced platform for acoustofluidics to enhance the impact of both acoustic wave propagation and localized heating transfer. The attached droplets are easily activated, jetted, or evaporated^24^ before the ice nucleation happens, thus significantly preventing or delaying ice formation and accumulation.

When ice crystals are attached to the device surface, similar interfacial reactions which have been discussed in the de-icing process should happen at the interfaces between the ice and the surface. The structures of the surface ice crystals will be broken due to the surface vibrations, while the thermo-heating effect will promote the phase changes and melt the ice into the liquid. The acoustic pressure/forces inside the liquid promote the exchanges of mass and heat which effectively prevent ice formation.

## 2 Method

### 2.1 Preparation of the SAW Device

A ZnO film of ∼5 μm thickness was deposited on 1.5 mm-thick Al plates using the DC magnetic sputtering technique. A zinc target with 99.99% purity was used during the deposition. The DC power was 400 W, and the Ar/O_2_ gas flow was 10/15 (in the unit of sccm). The crystalline structure of the ZnO film was analyzed using an X-ray diffractometer (XRD, D5000, Siemens) with Ni-filtered Cu Kα radiation (40 kV, 30 mA and λ = 1.5406 Å). The XRD pattern of the ZnO film on the aluminum substrate shows a strong peak of the ZnO (0002) diffraction plane. This indicates the *c*-axis preferential growth of the Wurtzite-structure ZnO film. The interdigital transducers (IDTs) were patterned on top of the ZnO thin film using a conventional photolithography and lift-off process. A bilayer of Cr/Au with a thicknesses of 20/100 nm was prepared using a thermal evaporator (EDWARDS AUTO306) as the electrode. The IDTs were designed with a wavelength of 400 μm, comprising 30 pairs of electrodes. The corresponding Rayleigh wave frequency measured using a network analyzer was 7.22 MHz.

### 2.2 Icing and Anti-Icing Process

To create a stable and constant icing/anti-icing environment, the experiment was conducted in a freezing chamber that was built based on a cold plate (Para Cooler A, Para Cooler O, Weinkauf Medizintechnik, Germany) with a sealed resin shield. The accurate humidity was achieved using an atomizer (Omron Ultrasonic Nebulizer NE-U17) that generated water aerosols with controlled vaporizing power and imputing speed. The velocity of airflow (∼3.8 m/s) and temperature consistency inside the chamber was stabilized using an electric fan. The cold plate was set at −6.5 °C, which kept the environmental temperature at −1 °C and the device temperature at −10 °C. Before the start of icing, the SAW device was cooled down in the chamber for 20 min in advance. Then, the icing process was carried out with RH levels of 60, 70, 80%, and 90%, respectively. The anti-icing study was performed with different RH levels and SAW powers (from 0.002 to 2.300 W). The icing duration lasted for 20 min while an IDS camera with a Navitar 12× objective lens was used to record the ice morphology from the top and side of the device. The mass of rime ice accumulated was measured after the icing process. Each test was repeated three times to get the average value. An infrared camera was used to monitor temperature changes of the SAW device surface with a humidity of 25% in the same chamber with the same airflow speed.

### 2.3 De-Icing Process

To do the de-icing tests, all the samples were cooled down in the chamber with a temperature of −6.5 °C for 20 min. The de-icing was carried out (for 5 min at most) with various RH levels of 60, 70, 80, and 90%, respectively. The SAW reflection signal of S_11_ was measured every 1 min. After the ice formation, the SAW was applied with various powers (from 0.400 to 2.300 W with proper gaps) to evaluate the de-icing performance. A high-speed camera (HotShot 1280 CC) with a Navitar 6.0× zoom lens and a 1.5× objective lens and the IDS camera with a Navitar 12× objective lens were also used to record the de-icing process from the top and side view.

## 3 Results and Discussion

### 3.1 Device Characterization

The SAW device used in this study was formed on the ZnO thin film deposited on a 1.5 mm aluminum plate, and the designed wavelength was 400 μm with a measured Rayleigh resonant frequency of 7.22 MHz. The electromechanical coupling coefficient (*k*^2^) of the SAW device was ∼1.75%, whereas the temperature coefficient of frequency (TCF)^24^ was ∼248 ppm/°C (see the Supporting Information).

The reflection spectra S_11_ of the SAW device (measured at room temperature, sub-zero temperature, and after the icing with various humidity levels at subzone temperature) are shown in Figure S1 in the Supporting Information The results show that the ice accretion caused serious damping of SAW signals. All the necessary basic information about the SAW device used in this study is summarized in Table 1.

**Table 1:** Experimental Parameters of the SAW Device Used in This Study

parameters	values
materials of piezoelectric thin film	ZnO
materials of substrate	aluminum sheet with 1.5 mm thickness
wavelength	400 μm
frequency	7.22 MHz
electromechanical coupling coefficient (*k*^2^)	∼1.75%
TCF	∼248 ppm/°C

The thermal heating effect on the surface of the SAW device was characterized using an infrared camera. The obtained temperature changes of the SAW device surface with various SAW powers within 90 s in an ambient environment (17 °C) and in a cold chamber (−10 °C) with 25% humidity are summarized in Figure S2 in the Supporting Information. Selected infrared images are shown in Figure S3 in the Supporting Information. As expected, with the increase in the applied SAW power, the surface temperature increases. When the SAW power is applied continuously, the recorded temperature changes at a substrate temperature of −10 °C become more significant than those of the substrate at room temperature (as shown in Figure S2a,b). The obtained data are summarized in the Supporting Information.

### 3.2 Anti-Icing Performance under SAWs

**Figure 2:**
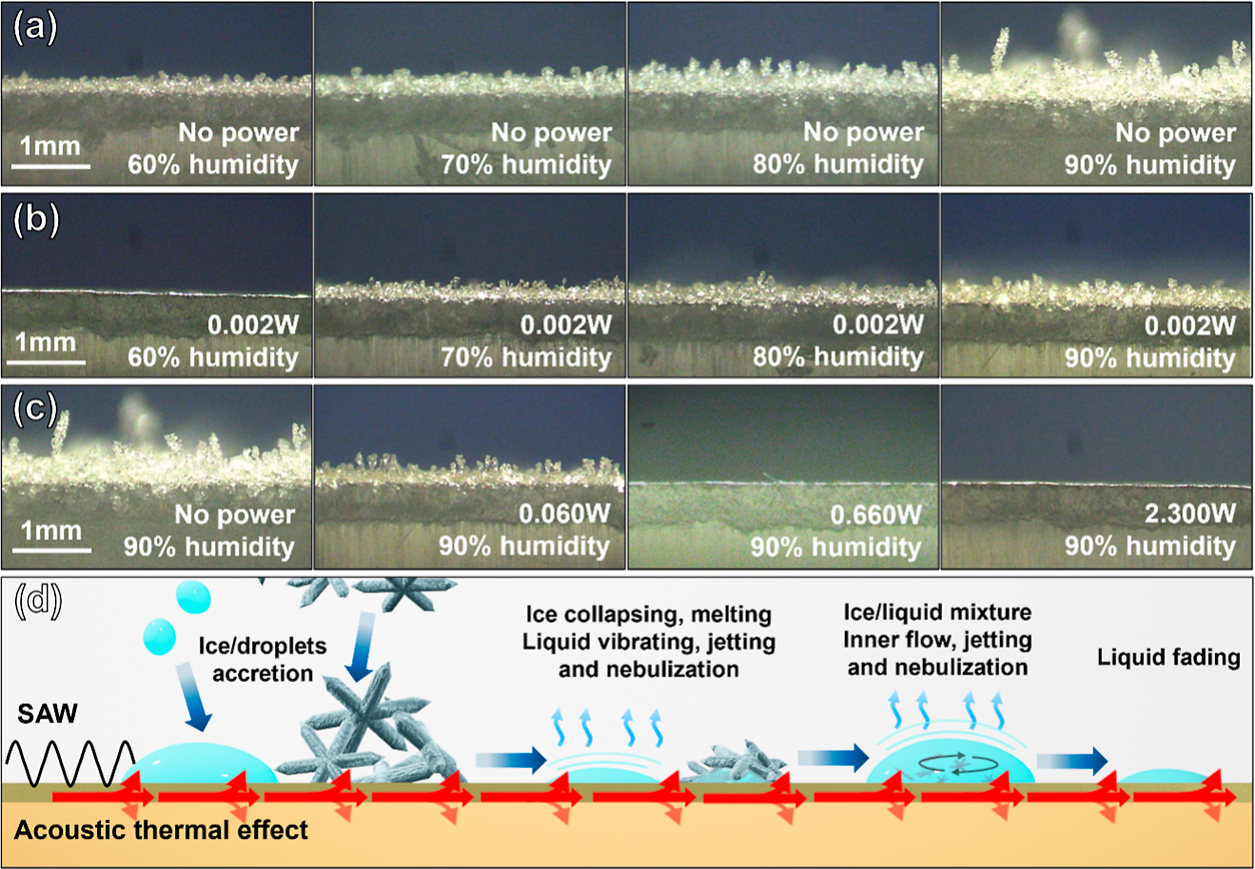
Cross-sectional surface or ice morphology after the 20 min icing process: (a) without applying SAW power at different humidity levels, (b) with the power of 0.07 W at different humidity levels, (c) with different SAW powers at 90% humidity, and (d) schematic anti-icing process and mechanism on the surface of the SAW device (including the ice/droplet accretion, ice collapsing, and melting and liquid inner flow, vibrating, jetting, nebulization, and then fading gradually).

Figure 2a–c shows the icing morphologies formed on the SAW device after 20 min, with various applied SAW powers at different RH levels. Figure 2a indicates that when there are no SAWs being applied, the surface ice layer becomes much thicker with the increase in the RH level as expected. The ice morphology is also changed from a thin ice layer at 60% humidity to the typical ice clusters and thick layer at 90% humidity.

Figure 2b shows the icing morphology after a SAW power of 0.002 W was applied at different RH levels. Similar to Figure 2a, the ice layer becomes much thicker, and the ice crystals grow much larger with the increase in the RH level. However, even at such a low power of 0.002 W, ice accretion is reduced effectively. There is no visible ice at 60% RH as shown in Figure 2a. At the other humidity levels, the thickness of the ice layer is much thinner and the size of ice clusters is smaller as shown in Figure 2a.

Figure 2c shows the icing morphologies in the environment with 90% RH at different SAW powers. Compared to Figure 2a without the applied power, the anti-icing effect is significant, even at a low power of 0.060 W, showing only a thinner ice layer and tiny ice crystals. When the power is increased to 0.660 and 2.300 W, the surface of the device does not show apparent icing phenomena. These results prove that ice accretion can be effectively restrained at low SAW powers.

**Figure 3:**
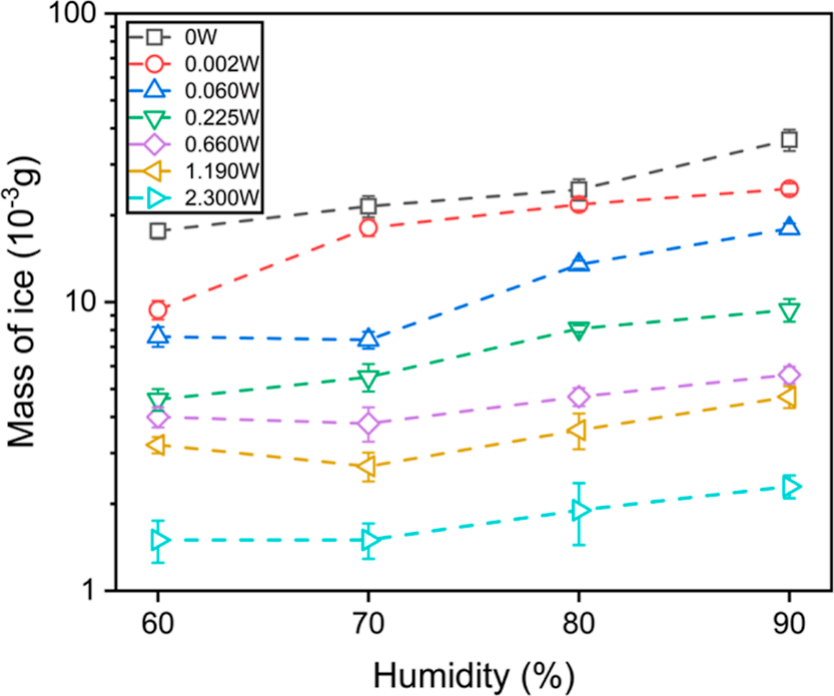
Estimated mass values of 20 min ice accretion on the 400 μm-wavelength SAW device with various humidity levels and SAW powers.

Figure 3 summarizes the increased mass values of ice accretion on the SAW device without and with various SAW powers at −10 °C. The mass of ice was calculated by weighing the device before and immediately after the icing process. Generally, the mass of ice is decreased with the increase in SAW power or the decrease in the humidity level. When there is no power applied, the ice buildup is around 17.65 to 36.50 mg with different humidity levels from 60 to 90%. Once a SAW is applied, even at extremely low powers such as 0.002 or 0.060 W, the mass of ice is decreased by several milligrams (3.20 to 11.80 mg, varied with different RH levels). When the SAW power is increased to 2.300 W, the ice buildup ranges from around 1.45 to 2.10 mg. Combining the results of Figures 2 and 3, it can be found that even at a relatively high power such as 2.30 W, there is always an increase in mass while the surface is dry and clean. The possible reason is that SAWs are generated from the IDT area and propagate along the surface, and they become slightly damped when they propagated far away from the IDT. The images shown in Figure 2 were recorded near the IDT area. The mass increases due to ice formation shown in Figure 3 are the results for the whole surface. Thus, the increase in mass of high powers was caused by the ice/droplet accretion in the area that was away far from the IDTs.

Based on the above results and discussion in Section 2, a schematic illustration of the anti-icing mechanism under the SAW actuation is illustrated in Figure 2d. When the arriving supercooled droplets are attached to the surface, the SAW induces internal flow by streaming force, while the acoustic thermal effect causes a local heating effect. The ice nucleation is restrained, and the exchange of the mass and heating inside the liquid is also enhanced by these two effects, which lead to further ice melting and liquid pumping, jetting or nebulization, or evaporation, which have been extensively reported in the literature.^27,58,59^ For the possibly attached ice crystals, the surface vibration induced by the acoustic wave agitates the attached areas of these ice crystals, which destroys their structures. At the ice/device interface, the acoustic thermal effect causes a local heating effect; thus, the ice crystals melt easily. The formation of a liquid layer provides a good medium to absorb SAWs, leading to the internal flow inside the liquid, as explained in Section 2. The further de-icing process is similar to the supercooled droplet route. The experimentally observed phenomena are consistent with those from the hypotheses in Section 2 and prove that the acoustic vibration and localized heating prevent ice nucleation and further accretion.

### 3.3 De-Icing Performance under SAWs

To perform de-icing tests, the rime ice was first formed at various RH levels for 20 min, and then, the SAW power was applied to study the de-icing phenomena. Table 2 lists the obtained de-icing times, which are defined as the durations to remove all the surface rime ice. When the power was low (e.g., 0.400 W), there were no visible changes in the ice morphology at all different RH levels. Similar phenomena were observed when the power was 0.660 W with high RH levels of 70 to 90%. The de-icing was not observed after 5 min, and these cases are labeled N/A as listed in Table 1. Generally, with the increase in SAW power and decrease in the RH level, the de-icing time is decreased systematically, whereas under certain conditions, results show the opposite trends, for example, the cases of 0.820 W at 90% humidity and 1.190 W at 60% humidity. A possible reason is that the porous structure of rime ice and the melted liquid lead to the uncontrollable damping of SAW energy, which cannot be prevented during the icing processes.

**Table 2:** De-Icing Time (in s) with Various Icing Humidity Levels and SAW Powers

humidity (%)	0.400 W	0.660 W	0.820 W	1.190 W	2.300 W
60	N/A	45.8 ± 5	7.9 ± 4.1	13.1 ± 2.8	9.5 ± 0.6
70	N/A	N/A	14.8 ± 1.7	17.3 ± 2.5	8.4 ± 0.4
80	N/A	N/A	24.0 ± 3.1	18.1 ± 1.6	7.2 ± 0.8
90	N/A	N/A	16.7 ± 2.0	21.0 ± 3.1	8.1 ± 0.7

**Figure 4:**
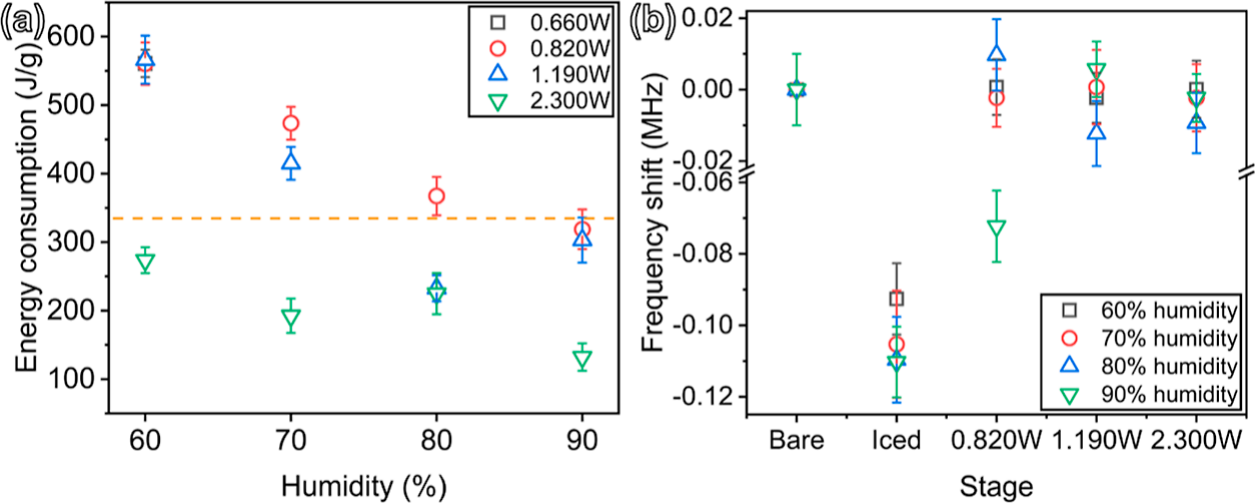
(a) Energy consumption of various de-icing processes and (b) resonant frequency shift at different icing and de-icing stages.

Based on the obtained results, the specific energy consumption for removing ice can be estimated using the following expression
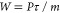
8where *P* is the SAW power, τ is the consumed time of removing the ice on the IDT area, and *m* is the mass of ice on the IDT area. Figure 4a summarizes the obtained data on energy consumption of the de-icing processes. The general trend observed is that with the increase of input SAW power, energy consumption is much smaller, corresponding to shorter de-icing times. The orange dash line in Figure 4a represents the enthalpy of fusion of ice, whose value is around 333.55 J/g.^60,61^ As it is well known, the conventionally used electrothermal de-icing technique generally has low energy efficiency and high energy consumption,^12,62^ whereas the SAW technology has its advantages such as the high efficiency for the de-icing process because of the combined effects of acoustic vibration and acoustic thermal effects, both of which are localized at the ice/device interface, as reported in Yang et al*.*^31^

Figure 4b shows the measured resonant frequency shifts of the SAW devices at different icing stages, which include the results for the surface in a sub-zero environment, iced surface with various RH levels, and de-iced surface with various de-icing powers and RH levels. After the 20 min icing process, the resonant frequency has been shifted by ∼0.1 MHz and the frequency shift becomes much larger with the increase in the RH level. After the de-icing process, the frequency shift is reduced to less than ∼0.01 MHz. Meanwhile, if the de-icing power is not high enough, the frequency shift remains a large value at a high humidity level, such as about 0.75 MHz with 0.820 W and 90% humidity.

**Figure 5:**
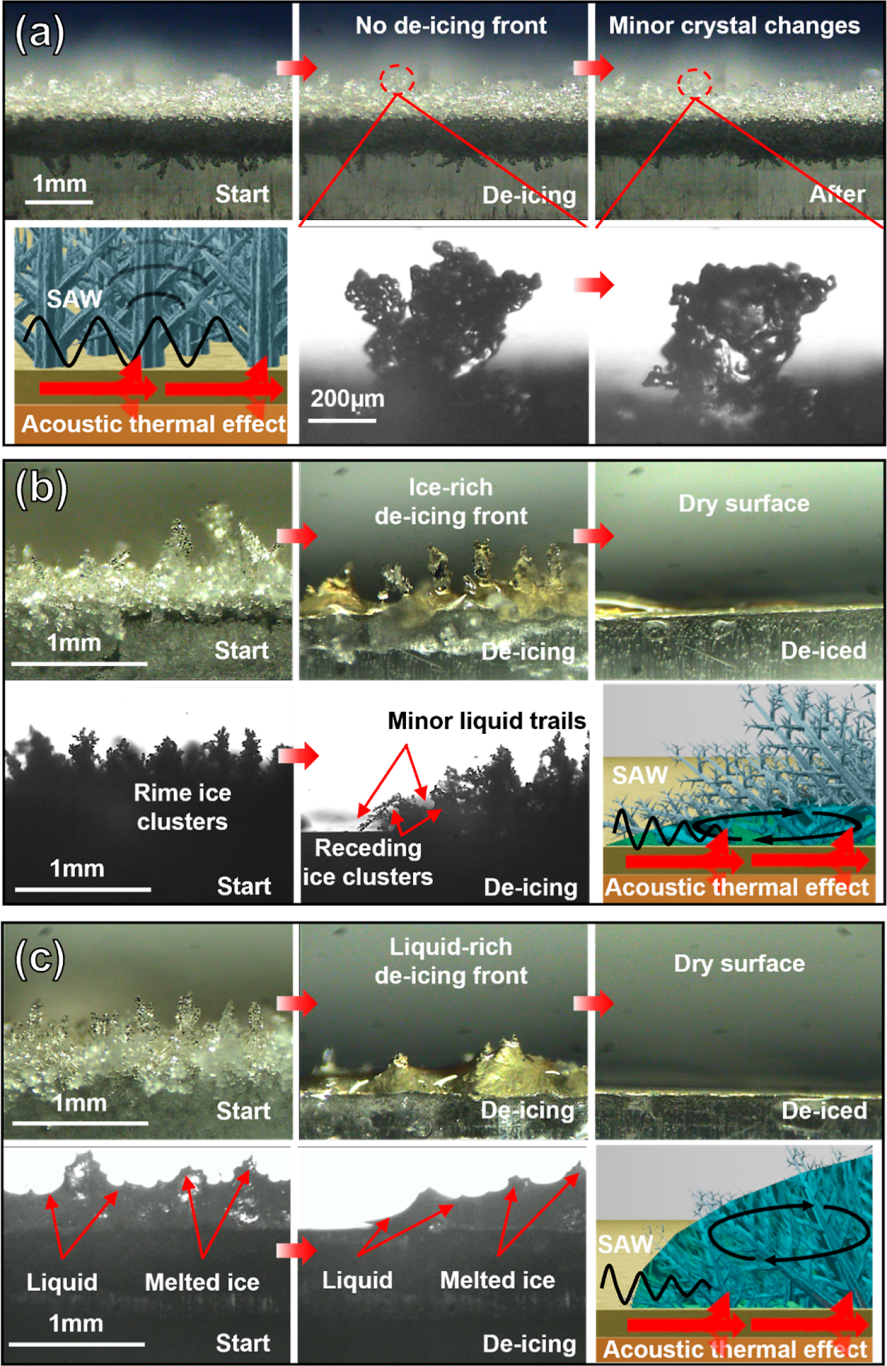
(a) Changes in ice morphology or ice crystals before the occurrence of possible phase changes with the propagation of SAW, (b) de-icing phenomenon of the ice-rich de-icing front, and (c) de-icing phenomenon of the liquid-rich de-icing front. (The colorful photographs were captured using an IDS camera with a Navitar 12× objective lens, while the black-and-white photographs were captured with a high-speed camera (HotShot 1280 CC) with a Navitar 6.0× zoom lens and 1.5× objective lens. The possible illustration of the propagation of SAWs at the interfaces was also drawn for different de-icing phenomena.)

Based on the experimental observations, the de-icing process can be summarized in Figure 5a–c. There are three major stages during the de-icing process that match well with the hypotheses explained in Section 2.

The first stage in Figure 5a shows that there are no significant visible changes in the ice morphology after the application of SAW powers. However, high-speed images (Figure 5a) clearly show that there are dramatic changes in ice clusters or single crystals, which are linked with a local collapse of ice clusters or fracture of the ice crystal structures caused by acoustic wave vibrations. At this initial stage, no apparent phase change (or melting) takes place.

The second stage in Figure 5b shows that the phase changes occur and the de-icing front appears. This de-icing front initially starts from the top position of the IDTs. At this stage, the acoustic vibration and thermal heating provide enough energy to the ice crystals to locally melt them into a liquid layer, which is then merged with the ice clusters. These large ice clusters are often seen to collapse into a thin liquid layer. Once the liquid phase appears, the acoustic streaming force (explained in eq 5) will drive the liquid into the porous ice layer. With the enhanced exchange of mass and heat induced by the acoustic streaming force, this liquid/ice crystal de-icing front is seen to gradually move along the direction of SAW’s propagation. At this stage, most of the ice clusters remain in their initial states, whereas some of them gradually collapse with the gradual moving of the de-icing front. At this step, ice clusters or crystals still occupy most of the de-icing front, which can be defined as the ice-rich front, while behind this ice-rich front, the liquid layer was quickly evaporated due to the SAW agitation.

With the further de-icing process, the third stage is formed as shown in Figure 5c. This stage occurs in zones that are often far away from the IDT area. At this stage, the de-icing front becomes much wider. The ice crystals shrink significantly without clear ice cluster morphologies. These semi-melted ice crystals are dispersed in the liquid and driven by acoustic radiation force (eq 6) and the acoustic streaming drag force (eq 7) based on the size ranges. The melting becomes significant, and the liquid layer becomes more apparent, which can form a liquid-like front in front of the remaining rime ice. This liquid layer absorbs SAW energy and causes a serious damping effect of the SAW signals. Meanwhile, the acoustic streaming inside the liquid enhances the heat transfer and also causes significant pumping, jetting, or nebulization effects,^24^ which we have observed using the high-speed camera. These two effects lead to the faster conduction of the thermal effect than that of acoustic waves in the area away from the IDT region. The mixed front with ice and water had been quickly moved along the surface until all the ice disappeared, with the remaining liquid vibrating, nebulizing, or evaporating on the device’s surface (see the videos about the de-icing process in the Supporting Information).

**Figure 6:**
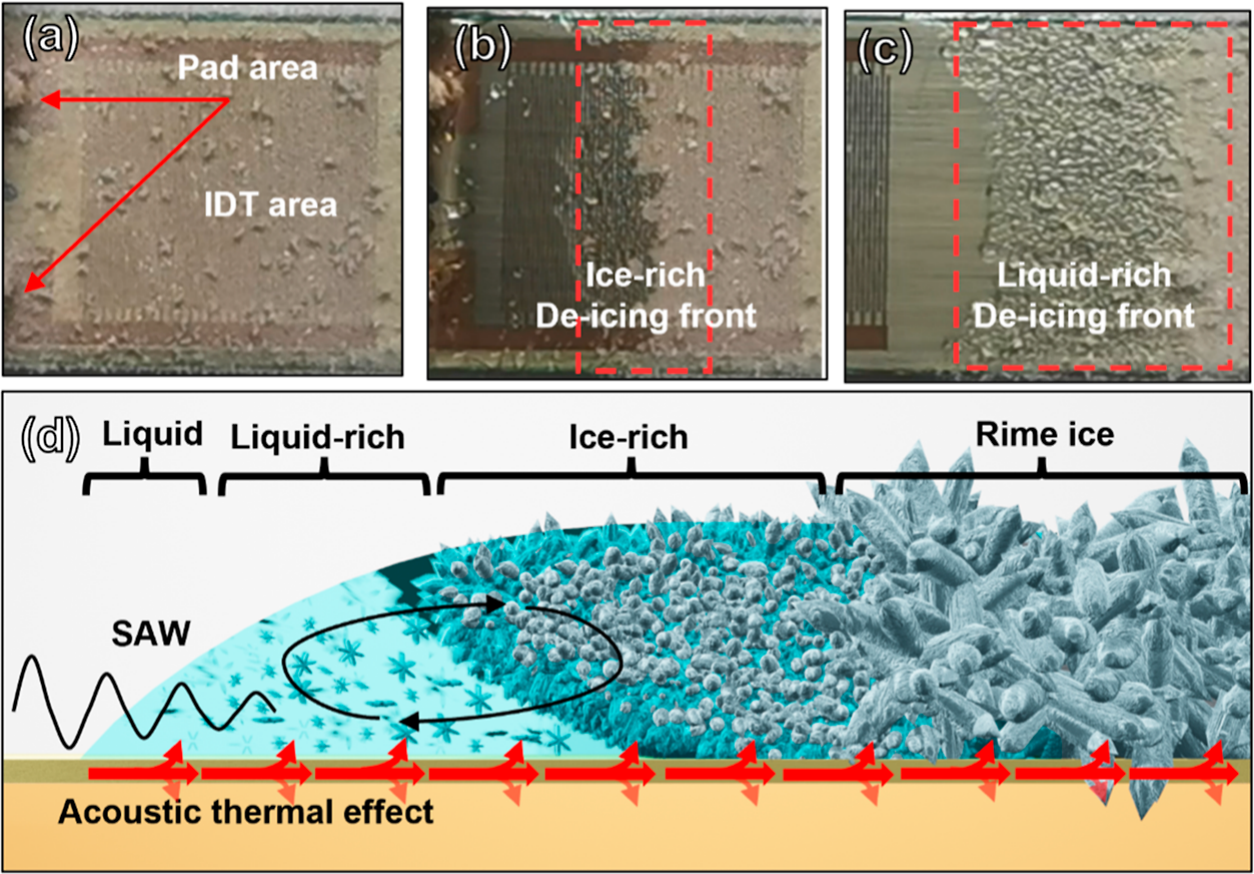
(a) Surface morphology of the iced SAW device (in the IDT area, top view), (b) area with an ice-rich de-icing front, (c) area with a liquid-rich de-icing front, and (d) schematic illustration of the de-icing front with four regions.

Figure 6a–c shows the enlarged de-icing fronts on the SAW device during three stages of the de-icing process. Before the phase changes occur, due to the large differences in acoustic impedance of the porous ice layer with the substrate, the acoustic energy of SAWs is significantly dissipated but confined into the rime ice. This causes significant vibrations of the porous structure, thus leading to plenty of local changes in porous rime ice and its surface crystals.

With further SAW agitation, phase changes happen, starting near the top area around the IDT. An icing front consisting of a liquid/ice mixture is formed and quickly moves along the SAW direction under the hybrid action of the acoustic wave and thermal heating effects. The width of the de-icing front is enlarged obviously with the continuation of the de-icing process (from Figure 6b to Figure 6c). The de-icing front can be classified into four areas that are the liquid area, the liquid-rich (with ice) area, ice-rich (with liquid) area, and the rime ice area, as shown in Figure 6d. In the liquid and liquid-rich areas, the SAW wave energy is efficiently absorbed due to low acoustic impedance, thus leading to significant internal streaming and phenomena of liquid transportation, jetting, or nebulization. Some small ice crystals can be seen flowing inside the liquid, stimulated by the acoustic radiation force and the acoustic streaming drag force. In the liquid-rich and ice-rich areas, acoustic wave energy is quickly dissipated into the liquid, causing both local heating and significant streaming effects. The acoustic streaming enhances the exchange of mass and heat, which accelerates the de-icing process. In the rime ice area, several events occur including the breakup and collapse of ice crystals or clusters, in the interfacial region between the substrate and rime ice.

In brief, we have confirmed that the key de-icing mechanisms are the phase changes induced by the hybrid effect of acoustic waves and the thermal effect. The surface vibrations induced by the acoustic waves affect the interfacial structures between the rime ice and the device, leading to the breakup and collapse of ice crystals or clusters. With the accumulation of the thermal effect, phase change also occurs at the ice/device interface, and the melted liquid and the ice crystals are quickly merged. Apart from the above two major effects, when the SAW waves propagate into this ice–liquid mixture front, the inner flow and streaming force significantly enhance the exchange of mass and heat, which effectively promotes the de-icing process.

## 4 Conclusions

In this study, anti-/de-icing mechanisms of rime ice using thin-film SAW technology were studied systematically. Anti-icing results showed that the anti-icing performance was improved significantly with the increase in the SAW power, and de-icing results showed that the de-icing energy efficiency was quite high for the SAW device, even in a severely frozen environment at a high humidity level. On comparing with the potential thermal energy consumption of thermal melting ice, both acoustic wave vibration and acoustic heating play key roles during the de-icing process. The surface vibration induced the breakup and collapse of ice crystals or clusters. The accumulation of heat prompted the melted liquid that absorbed the SAW waves and led to the internal streaming to enhance the exchange of mass and heat. These effects generated further ice melting, liquid pumping, jetting or nebulization, and evaporation during the icing process, while the formation and movement of the de-icing front in the de-icing process were enhanced accordingly.

## Supporting Information Available

Equation S1-S2, reflection (S11) spectra of thin film SAW device, measured temperature changes on the device surface, and device surface temperature applying SAW power at 0s and 80s(PDF)
De-icing process with 80% icing humidity and 0.400 W de-icing power (MP4)
De-icing process with 80% icing humidity and 0.660 W de-icing power (MP4)
De-icing process with 80% icing humidity and 0.820 W de-icing power (MP4)
De-icing process with 80% icing humidity and 1.190 W de-icing power (MP4)
De-icing process with 80% icing humidity and 2.300 W de-icing power (MP4)

## Abbreviations

SAW: surface acoustic wave
RH: relative humidity
TCF: temperature coefficient of frequency
IDT: interdigital transducerss
