# Supplementary material for: Dynamic Mitigation Mechanisms of Rime Icing with Propagating Surface Acoustic Waves
Source: Langmuir. 2022 Sep 7;38(37):11314–23. doi: 10.1021/acs.langmuir.2c01509 (PMC9494940; doi:10.1021/acs.langmuir.2c01509)
Supplement: Supplementary file 1 — la2c01509_si_001.pdf [file la2c01509_si_001.pdf]

Supporting Information

Dynamic mitigation mechanisms of rime icing with propagating surface acoustic waves

Deyu Yang,[1] Luke Haworth,[2] Prashant Agrawal,[2] Ran Tao,[3] Glen McHale,[4] Hamdi Torun,[2] James Martin,[2] Jingting Luo,[3] Xianghui Hou,[1,] YongQing Fu [2,*]*

[1]Faculty of Engineering, University of Nottingham, Nottingham, NG7 2RD, UK

[2]Faculty of Engineering and Environment, Northumbria University, Newcastle upon Tyne, NE1 8ST, UK

[3]Shenzhen Key Laboratory of Advanced Thin Films and Applications, College of Physics and Optoelectronic Engineering, Shenzhen University, Shenzhen, 518060, China

[4]School of Engineering, University of Edinburgh, Edinburgh, EH9 3JL, UK

*Corresponding Author: Dr. Xianghui Hou, E-mail: xianghui.hou@nottingham.ac.uk, Prof. Richard Yongqing Fu, E-mail: richard.fu@northumbria.ac.uk

Table of Content

Equation S1	page S3
Equation S2	page S3
Figure S1	page S4
Figure S2	page S5
Figure S3	page S6
Video S1	page S7
Video S2	page S7
Video S3	page S7
Video S4	page S7
Video S5	page S7

Equations S1-S2:

Electromechanical coupling coefficient (k^2) and temperature coefficient of frequency (TCF) of the device were obtained using [1]:

$$k^2 = \frac{\pi}{4N}\left(\frac{G}{B}\right)_{f=f_0} \quad \text{(S1)}$$

$$TCF = \frac{1}{f_0}\frac{df}{dT}(ppm/°C) \quad \text{(S2)}$$

where N is the finger pairs of the IDTs; G and B are the radiation conductance and susceptance of the input port, obtained from the Smith charts of the reflection coefficients at the central frequency (f_0) of the SAW signals. The TCF value was obtained based on the shifts of the resonant frequencies within a temperature range from 30°C to 80°C.

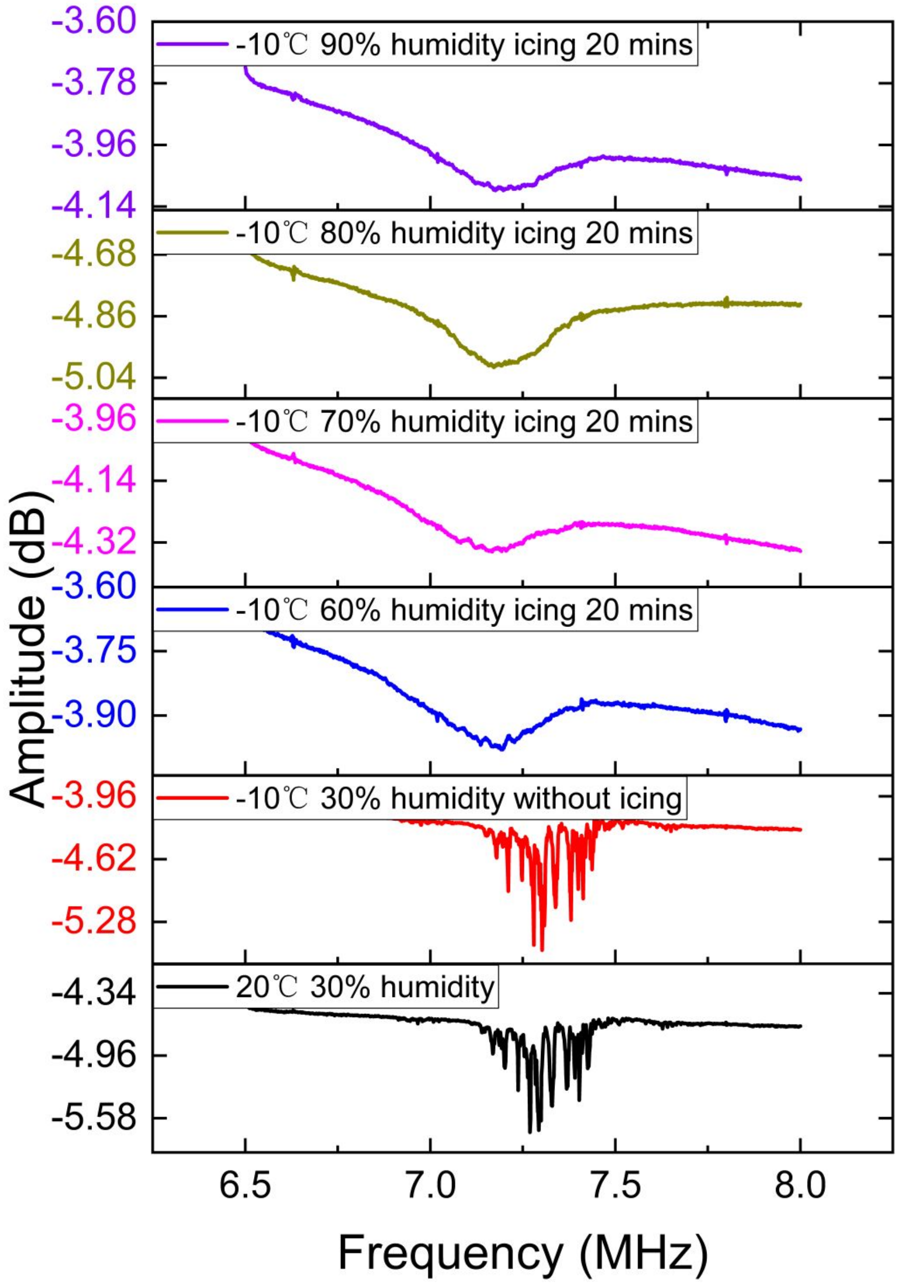

Figure.S1 Reflection (S_{11}) spectra of thin film SAW device with a wavelength of 400 μm, obtained at room temperature, subzero temperature (-10℃), and after 20-minute icing duration at different relative humidities

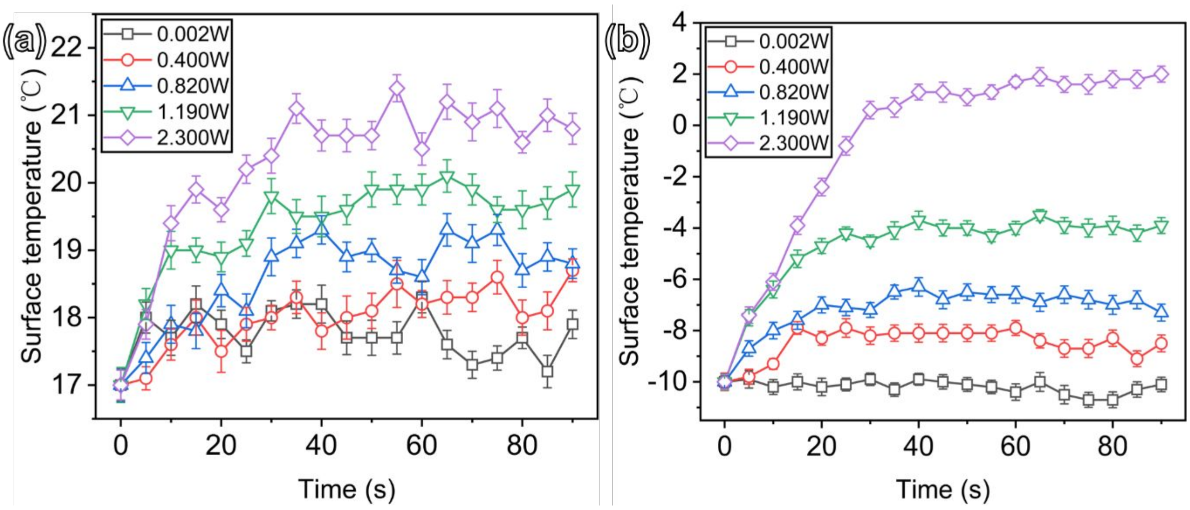

Figure S2. Measured temperature changes on the device surface under various SAW powers with a duration of ~90 seconds at two different testing substrate temperatures with the humidity of 25%: (a) room temperature; (b) -10℃.

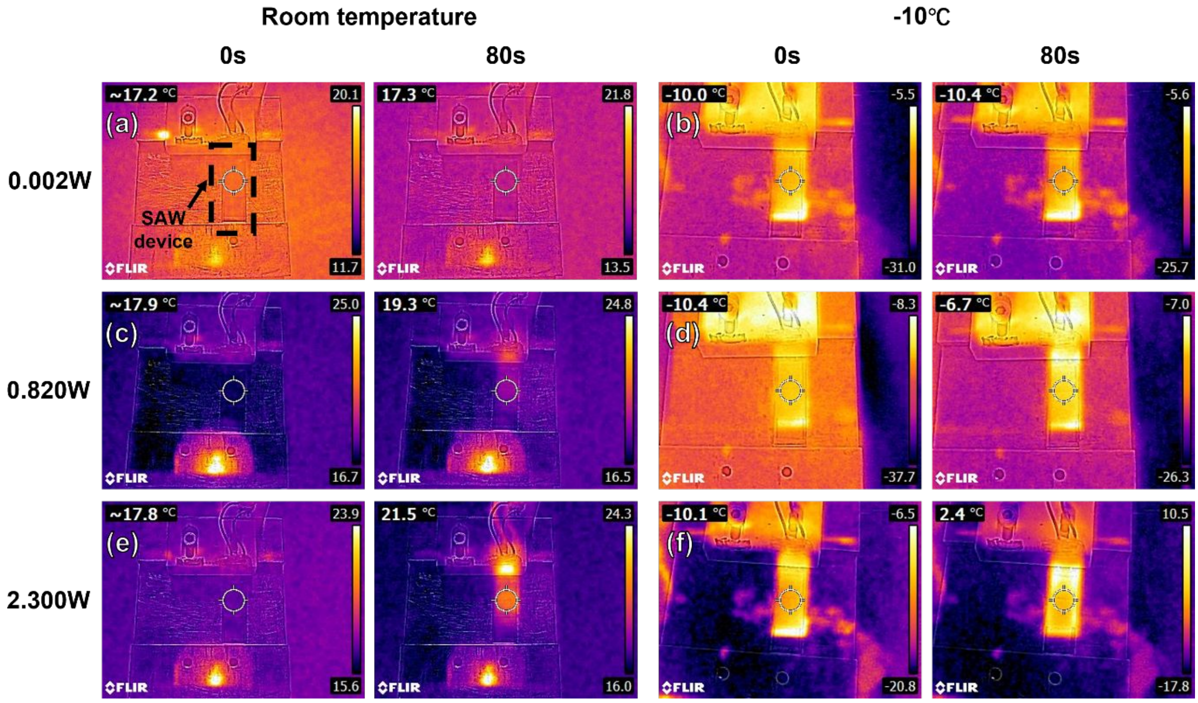

Figure S3. The device surface temperature applying SAW power at 0s and 80s in the environment with 25% humidity : (a) room temperature, 0.002W SAW power; (b) -10°C 0.002W SAW power; (c) room temperature, 0.820W SAW power; (d) -10℃ 0.820W SAW power; (e) room temperature, 2.300W SAW power; (f) -10℃ 2.300W SAW power;

Video S1: De-icing process with 80% icing humidity and 0.400 W de-icing power.

Video S2: De-icing process with 80% icing humidity and 0.660 W de-icing power.

Video S3: De-icing process with 80% icing humidity and 0.820 W de-icing power.

Video S4: De-icing process with 80% icing humidity and 1.190 W de-icing power.

Video S5: De-icing process with 80% icing humidity and 2.300 W de-icing power.

References

1. Fu, Y. Q.; Luo, J.; Nguyen, N.-T.; Walton, A.; Flewitt, A. J.; Zu, X.-T.; Li, Y.; McHale, G.; Matthews, A.; Iborra, E., Advances in piezoelectric thin films for acoustic biosensors, acoustofluidics and lab-on-chip applications. *Prog Mater Sci* **2017,** *89*, 31-91.
